# Central Roles and Regulatory Mechanisms of Dual-Specificity MAPK Phosphatases in Developmental and Stress Signaling

**DOI:** 10.3389/fpls.2018.01697

**Published:** 2018-11-20

**Authors:** Lingyan Jiang, Yinhua Chen, Lijuan Luo, Scott C. Peck

**Affiliations:** ^1^Hainan Key Laboratory for Sustainable Utilization of Tropical Bioresource, Hainan University, Haikou, China; ^2^Institute of Tropical Agriculture and Forestry, Hainan University, Haikou, China; ^3^Department of Biochemistry, University of Missouri, Columbia, MO, United States; ^4^Christopher S Bond Life Sciences Center, University of Missouri, Columbia, MO, United States; ^5^Interdisciplinary Plant Group, University of Missouri, Columbia, MO, United States

**Keywords:** mitogen-activated protein kinase, phosphatase, regulatory mechanism, development, stress signaling

## Abstract

Mitogen-Activated Protein Kinase (MAPK) cascades are conserved signaling modules that integrate multiple signaling pathways. One level of control on the activity of MAPKs is through their negative regulators, MAPK phosphatases (MKPs). Therefore, MKPs also play an integrative role for plants responding to diverse environmental stimulus; but the mechanism(s) by which these phosphatases contribute to specific signals remains largely unknown. In this review, we summarize recent advances in characterizing the biological functions of a sub-class of MKPs, dual-specificity phosphatases (DSPs), ranging from controlling plant growth and development to modulating stress adaptation. We also discuss putative regulatory mechanisms of DSP-type MKPs, which plants may use to control the correct level of responses at the right place and time. We highlight insights into potential regulation of cross-talk between different signaling pathways, facilitating the development of strategies for targeting such cross-talk and to help improve plant resistance against adverse environmental conditions without affecting the growth and development.

## Introduction

To rapidly adapt to various environmental challenges, plants need to balance diverse signal inputs which involves crosstalk between different signaling pathways. A common strategy for integrating these signals is through use of shared components, including mitogen-activated protein kinase (MAPK) cascades that play essential roles in signal transduction and amplification for many distinct cellular responses. MAPK cascade modules are conserved in all eukaryotes and consist of three kinase components: MAPK kinase kinases (MAPKKKs) phosphorylate dual-specificity MAPK kinases (MAPKKs), which then phosphorylate and activate the terminal MAPKs in a Thr-X-Tyr activation loop (Widmann et al., 1999; Bartels et al., 2010). The Arabidopsis genome encodes 20 MAPKs, 10 MAPKKs, and 60 MAPKKKs (Ichimura et al., 2002; Hamel et al., 2006); and several complete MAPK cascades in plants have been described (Colcombet and Hirt, 2008).

Because the same MAPK signaling modules can be activated by multiple extracellular stimuli, controlling the intensity and duration of MAPK activation is a critical determinant for organisms to generate correct biological outcomes for these different inputs. Therefore, an important point of regulating signaling is through dephosphorylation of MAPKs to attenuate their activity. Dephosphorylation of either the threonine and/or tyrosine residue within the activation motif inactivates MAPKs; and this dephosphorylation can be regulated by diverse types of protein phosphatases, including phosphoprotein phosphatases (PPPs) and metal-dependent protein phosphatases (PPMs), which are serine/threonine phosphatases, and tyrosine phosphatases (PTPs) (Shi, 2009; Bartels et al., 2010; Uhrig et al., 2013; Shankar et al., 2015). Representative members of the PPP family include protein phosphatase PP1, PP2A, PP2B, PP4, PP5, PP6, PP7; and the PPM family includes PP2Cs (Shi, 2009). These protein phosphatases likely have a variety of additional substrates, but some of PP2C-types have been shown to play important roles in regulating pathogen-associated signaling and defense responses at least partially through their control of MAPK activity (Meskiene et al., 2003; Schweighofer et al., 2007; Brock et al., 2010; Galletti et al., 2011; Sidonskaya et al., 2016; Mine et al., 2017). In support of potential integrative roles for MAPK-regulating phosphatases, the diverse functions of PP2Cs in plant hormone signaling, plant immunity, nutrition deficiency and development have been reviewed elsewhere (Fuchs et al., 2012; Singh et al., 2016).

PTPs are biochemically distinct protein phosphatases and include tyrosine specific phosphatases and dual-specificity (Ser/Thr and Tyr) phosphatases (DSPs) (Shankar et al., 2015). DSP-type MKPs are involved in diverse developmental processes and/or environmental stresses, ranging from salinity, drought, and UV-B radiation to pathogens (Gupta et al., 1998; Ulm et al., 2001, 2002; Lee and Ellis, 2007; Bartels et al., 2009; Lee et al., 2009; Walia et al., 2009; Anderson et al., 2011, 2014). In addition, most DSP-type MKPs in plants have several non-catalytic domains with the potential to interact with components of MAPK-independent signaling pathways (Bartels et al., 2010). These additional domains indicate that DSP-type MKPs are likely to coordinate different responses.

Because information on this small subset of protein phosphatases with diverse roles in plant biology has not been collated, the purpose of this review is to summarize recent progress in investigating and understanding the physiological roles of DSP-type MKPs in regulating multiple biological processes. We also discuss the putative regulatory mechanisms of DSP-type MKPs, which likely adds regulatory complexity for MKPs to orchestrate crosstalk between different signaling pathways.

## Dual specificity MAPK phosphatases as negative regulators of MAPK signaling

MAPK signaling cascades are essential components for regulating multiple cellular responses in eukaryotes. Once signals are initiated, they also need to be attenuated to prevent over-activation and to reset the system to basal levels after the initial stimulation. Phosphatases are important negative regulators of MAPK signaling. Among these, dual-specificity (Ser/Thr and Tyr) phosphatases (DSPs) belong to a subfamily of the tyrosine phosphatases (PTPs) that can dephosphorylate both phosphoserine/phosphothreonine and phosphotyrosine within the activation loop of MAPKs (Keyse and Emslie, 1992; Alessi et al., 1993; Sun et al., 1993; Ward et al., 1994). Twenty-two DSPs have been identified in Arabidopsis genome (Kerk et al., 2008), five of which have been shown experimentally to interact with and dephosphorylate MAPKs and, therefore, form the group of functional DSP-type MKPs (hereafter referred to in this review as MKPs) in Arabidopsis (Gupta et al., 1998; Ulm et al., 2001, 2002; Lee and Ellis, 2007; Lee et al., 2009; Walia et al., 2009). These five MKPs include DUAL-SPECIFICTY PROTEIN TYROSINE PHOSPHATASE 1 (DsPTP1), MAP KINASE PHOSPHATASE 1 (MKP1), MAP KINASE PHOSPHATASE 2 (MKP2), INDOLE-3-BUTYRIC ACID RESPONSE 5 (IBR5), and PROPYZAMIDE HYPERSENSITIVE 1 (PHS1).

*In vitro* and/or *in vivo* studies have shown that these five MKPs physically interact with MAPKs and/or regulate their activation (Table 1). DsPTP1 was the first dual-specificity protein phosphatase from higher plants shown to inactivate a MAPK (MPK4) *in vitro* (Gupta et al., 1998). MKP1 physically interacts with MPK3, MPK4, and MPK6 both *in vitro* and *in vivo*; and it deactivates MPK6 in protoplast-based assays (Ulm et al., 2002; Bartels et al., 2009). MKP2, which interacts with MPK3 and MPK6 both *in vitro* and *in vivo*, is able to dephosphorylate phospho-MPK3 and phospho-MPK6 *in vitro* (Lee and Ellis, 2007). IBR5, a MPK12 interacting partner *in vitro* and *in vivo*, has been shown to dephosphorylate and thus deactivate MPK12 (Lee et al., 2009). PHS1 interacts with Arabidopsis MAPKs MPK12 and MPK18 in a yeast two-hybrid interaction assay, and recombinant PHS1 dephosphorylates the activated MPK18 *in vitro* (Walia et al., 2009).

**Table 1:** Functions of DSP-type MAPK phosphatases in plants.

	**Regulatory mechanism**	**Interacting partners**	**Phosphatase substrates**	**Predominant subcellular localization**	**Biological roles**	**References**
DsPTP1	Transcriptional induction by osmotic stress; Catalytic activation by CaM binding *in vitro*	MPK4 (*in vitro*); CaM (*in vitro*)	pNPP, pMBP, MPK4	N.A.	Osmotic stress (−); ABA responses (+)	Gupta et al., 1998 Yoo et al., 2004 Liu et al., 2015b
MKP2	Catalytic activation by MPK3 and MPK6 *in vitro*	MPK3 (*in vitro, in vivo*); MPK6 (*in vitro*, *In vivo*)	OMFP, MPK3, MPK6	Nucleus (Arabidopsis transgenic plants); Nucleus and cytoplasm (Transient expression in tobacco cells)	Oxidative stress (+); Pathogen resistance	Lee and Ellis, 2007 Lumbreras et al., 2010
IBR5	Alternative splicing	MPK12 *(in vitro, in vivo*)	OMFP, MPK12	Nucleus (Arabidopsis transgenic plants driven by CaMV 35S promoter); Nucleus and cytoplasm (Arabidopsis transgenic plants driven by native promoter)	Auxin responses (+); ABA responses (+) Resistance protein (CHS3, SNC1, RPS4, RPM1)	Monroe-Augustus et al., 2003 Strader et al., 2008a Strader et al., 2008b Lee et al., 2009 Strader and Bartel, 2009 Jayaweera et al., 2014 Johnson et al., 2015 Liu et al., 2015a
OsIBR5	Transcriptional induction by PEG6000, ABA and H_2_O_2_	SIPK (*in vitro*); WIPK (*in vitro*); NTF3 (*in vitro*); MEK1 (*in vitro*)	N.A.	Cytoplasm and nucleus (Transient expression in rice protoplasts)	Drought/osmotic stress (−)	Li et al., 2012
PHS1	Transcriptional induction by ABA	MPK12 (*in vitro*) MPK18 (*in vitro, in vivo*)	OMFP, MPK18	Cytoplasm (Arabidopsis transgenic plants)	Cortical microtubule organization (+); ABA responses (−); Flowering (+)	Naoi and Hashimoto, 2004 Quettier et al., 2006 Walia et al., 2009 Pytela et al., 2010; Fujita et al., 2013; Tang et al., 2016
MKP1	Catalytic activation by phosphorylation; Catalytic activation by CaM binding; Increased protein stability by phosphorylation	MPK3 (*in vitro, in vivo*); MPK4 (*in vitro*) MPK6 (*in vitro, in vivo*) CaM (*in vitro*)	OMFP, MPK6	Cytoplasm (Arabidopsis transgenic plants)	Genotoxic resistance (+); Pathogen resistance (−); Salt resistance (−); Stomata development (+)	Ulm et al., 2001; Ulm et al., 2002; Lee et al., 2008; Bartels et al., 2009; Anderson et al., 2011; Park et al., 2011; González Besteiro and Ulm, 2013; Anderson et al., 2014; Jiang et al., 2017b; Tamnanloo et al., 2018
NtMKP1	Transcriptional induction by wounding and TMV-induced cell death; Catalytic activation by SIPK	SIPK (*in vitro*); CaM (*in vitro*)	OMFP, SIPK	N.A.	Wounding (−); Cell death (−); Pathogen resistance (−)	Yamakawa et al., 2004; Katou et al., 2005; Oka et al., 2013
TMKP1	Transcriptional regulation by salt and osmotic stress; Catalytic activation by 14-3-3 protein; Catalytic activation by TMKP3	CaM (*in vitro);* TMPK3 (*in vivo*); TMPK6 (*in vivo*)	OMFP	Nucleus (Transient expression in tobacco cells)	Salt stress (+)	Zaïdi et al., 2010; Ghorbel et al., 2015; Zaidi et al., 2016; Ghorbel et al., 2017
OsMKP1	Transcriptional induction by wounding	CaM (*in vitro*)	N.A.	N.A.	Wounding (−)	Katou et al., 2007

*NA, not available; −, negative regulator; +, positive regulator; OMFP, 3-O-methylfluorescein; pMBP, phosphorylated myelin basic protein; pNPP, para-nitrophenyl phosphate; TMV, tobacco mosaic virus*.

Supporting a role for these proteins as negative regulators of MAPK activation, MKP genetic mutants often showed prolonged and/or hyper-activation of MAPKs following stimulation; and a secondary mutation in or knock down of MAPKs in the background of the cognate interacting MKP mutant partially or completely suppressed the phenotypes of MKP mutants. For instance, both genotoxic stress and pathogen challenge treatments result in hyper-activation of MPK3 and MPK6 in the *mkp1* null mutant compared to wild type (Ulm et al., 2002; Anderson et al., 2011). Interestingly, loss of *MPK6* alone reverted a subset of *mkp1*-dependent phenotypes in a *mkp1 mpk6* double mutant, including the pathogen resistance, pathogen induced seedling growth inhibition and transcriptional variations (Anderson et al., 2011, 2014; Jiang et al., 2017b). A similar scenario has also been shown in the *mkp2*-RNAi mutant which is associated with a prolonged MPK3 and MPK6 activation during ozone treatment (Lee and Ellis, 2007). In addition, a knock down of MPK12 by RNAi in *ibr5* mutants partially reverted the auxin-insensitivity phenotype of *ibr5*; and a *phs1 mpk18* double mutant shows partial suppression of the *phs1-1* phenotypes (Lee et al., 2009; Walia et al., 2009). Therefore, building upon the biochemical data, genetic interactions between MKPs and their interacting MAPKs also are clearly established.

## Dual specificity MAPK phosphatases are regulators of plant growth and development

MAP kinase phosphatase IBR5 is a positive regulator of auxin responses, demonstrating a role for MKPs as coordinators of plant growth and development. The first evidence came from the observation that root growth of the *ibr5-1* mutant was more resistant to inhibitory concentrations of endogenous auxins (i.e., indole-3-butyric acid and indole-3-acetic acid), synthetic auxins and auxin transport inhibitors (Monroe-Augustus et al., 2003). The double mutant combination of *ibr5-1* with an auxin receptor mutant, *tir1*, displayed more extreme auxin resistance compared with either single mutant, suggesting that IBR5 regulates TIR1-mediated auxin signaling pathways (Strader et al., 2008a). To investigate potential mechanisms underlying the enhanced auxin resistance in the *ibr5* mutant, the accumulation of DR5::GUS, the GUS reporter driven by an auxin-responsive promoter, were measured after auxin treatments; and the *ibr5* mutant showed reduced induction of DR5::GUS in both roots and hypocotyls (Strader et al., 2008a). Many Aux/IAA repressor proteins directly prevent transcriptional activation induced by auxin treatments (Tiwari et al., 2003, 2004). Furthermore, stabilization of Aux/IAA repressor proteins were observed in numerous other auxin-resistant mutants, including *tir1* (Gray et al., 2001). Therefore, the accumulation of the Aux/IAA repressor proteins AxR3/IAA17 and IAA28 was investigated in the *ibr5* mutant. Interestingly, unlike other auxin-resistance mutants, both AxR3/IAA17 and IAA28 were not stabilized in *ibr5*, suggesting that IBR5 facilitates auxin responses independently and/or downstream of TIR1-mediated degradation of Aux/IAA repressor proteins (Strader et al., 2008a). Possible targets of IBR5-regulated pathways came from genetic screens for suppressors of *ibr5-1*. Suppressor mutations in *PLEIOTROPIC DRUG RESISTANCE 8 (PDR8)* and *PDR9* genes were characterized, and these two genes encode ABC transporters implicated in the efflux of synthetic auxins (Strader et al., 2008b; Strader and Bartel, 2009).

The mutation of *ibr5-1* causes a premature stop codon, resulting in a truncated protein lacking the conserved phosphatase domain (Monroe-Augustus et al., 2003). To examine the contribution of the phosphatase domain and/or phosphatase activity for the full function of IBR5, a mutant version of IBR5 lacking the active site cysteine was expressed in the background of *ibr5-1*; and the responses to auxin treatment were tested. Expression of the catalytically inactive version failed to fully complement the phenotypes of *ibr5-1*, suggesting that phosphatase activity is required for full IBR5 function (Strader et al., 2008a).

In addition to regulating root growth, IBR5 also controls the size of above ground organs. In *ibr5-6* mutants affecting the active site of the dual-specificity phosphatases, plants have reduced petal size and a smaller stature compared to wild type (Johnson et al., 2015). Microarray studies identified a set of genes associated with auxin synthesis, transport, regulation and responses that were mis-regulated in *ibr5-6* mutants compared to wild type (Johnson et al., 2015). These results support earlier studies that IBR5 regulates auxin signaling pathways and, in addition, also suggest that IBR5 may control the size and shape of petals through modulation of auxin signaling pathways.

Another area where MKPs have been shown to affect plant growth and development is in the control of the dynamics and organization of microtubules, in which PHS1 has been implicated to play an essential role. A semi-dominant *phs1-1* allele in *Arabidopsis thaliana* exhibits less ordered and slightly more fragmented cortical microtubule arrays in the roots of seedlings, and the epidermal cells of *phs1-1* roots are twisted in a left-handed helix under normal growth condition (Naoi and Hashimoto, 2004). When treated with low doses of microtubule-destabilizing drugs, the elongation of *phs1-1* roots were more severely inhibited; and the epidermal cells swelled, suggesting that the *phs1-1* mutants are hypersensitive to the microtubule-destabilizing drugs (Naoi and Hashimoto, 2004). Additionally, the *phs1-1* mutation exaggerates the temperature-sensitive phenotypes of *microtubule organization 1-1 (mor1-1)* mutants, which display a defect in a microtubule-associated protein (Whittington et al., 2001; Naoi and Hashimoto, 2004). Together, these results indicate that cortical microtubules are destabilized in the semi-dominant mutant *phs1-1*. Interestingly, other null mutant alleles of PHS1 are indistinguishable from wild type under standard growth conditions (Pytela et al., 2010).

In the *phs1-1* mutant, an Arg64 residue is replaced with Cys (R64C) in the putative kinase interaction motif (KIM) of MAPKs such that the mutation might interfere with interactions with the target MAPK(s). Therefore, it has been proposed that PHS1 may regulate multiple MAPKs; and a subset of its target kinases may be involved in the organization of cortical microtubules. To further investigate the mechanism underlying microtubule regulation, a screen for PHS1-interacting MAPKs was performed; and MPK12 and MPK18 were identified by yeast two-hybrid assays (Walia et al., 2009). Interestingly, *mpk18* seedlings also display defects in microtubule related functions; and a secondary mutation resulting in the loss of MPK18 in *phs1-1* partially complements the root growth phenotypes of *phs1-1* (Walia et al., 2009). This study provides support for the idea that PHS1 regulates the organization of microtubules through controlling the activity of the interacting MAPKs. If this hypothesis is correct, the phosphatase activity of PHS1 should play an essential role in this process. To explore this possibility further, a GFP fusion of the phosphatase-dead Cys792-to-Ser mutant (PHS1^C792S^-GFP) was expressed in the background of *phs1-5* to examine if the phosphatase-dead form of PHS1 functions as a dominant-negative microtubule destabilizer (Fujita et al., 2013). In transgenic seedlings, the organization of cortical microtubules was almost completely depolymerized. Additionally, a combination of the phosphatase-dead and R64C mutations results in substantially more depolymerization of cortical microtubules in the root epidermal cells than seen in the R64C mutation alone. These results indicate that the phosphatase inactive form of PHS1 triggers the destabilization of microtubule. One of the possible explanations for the destabilization caused by the phosphatase-dead form of PHS1 is that the PHS1 might have intrinsic microtubule destabilizing activity, which is normally suppressed by its phosphatase activity. To search for the putative region associated with potential microtubule destabilized activity, an examination of truncations of PHS1 has been performed, and an internal 69 kDa fragment lacking the phosphatase catalytic domain and KIM domain has been characterized (Fujita et al., 2013). Interestingly, this region has weak homology to the slime mold actin-fragmin kinase, which phosphorylates Thr349 of α-tubulin at the longitudinal inter-dimer interface, thereby generating a polymerization-incompetent isoform, and effectively depolymerizing microtubule arrays when expressed in plant and animal cells (Fujita et al., 2013). Such tubulin kinase activity may be suppressed by the phosphatase activity of PHS1 under normal growth condition, because α-tubulin is not normally phosphorylated in *Arabidopsis* plants grown in standard growth conditions; and transiently or stably expressed full-length PHS1 does not affect microtubule stability in *Arabidopsis* cells (Fujita et al., 2013). Also α-tubulin is not phosphorylated in the *phs1* null mutants; and expression of putative kinase domain of PHS1 results in phosphorylation of α-tubulin in the mutants (Fujita et al., 2013). As PHS1 interacts with and dephosphorylates MAPK18, it is possible that MAPK18 and/or other MAPKs may activate the tubulin kinase domain of PHS1, but that these MAPKs are normally inactivated by the phosphatase domain of PHS1.

In addition to controlling the microtubule organization, PHS1 is also involved in flowering time in Arabidopsis. The knock-out mutant *phs1-5* displayed a late flowering phenotype; and this alteration appears to result from altered expression of key genes in *phs1-5* (Tang et al., 2016). CONSTAN (CO) functions as an activator of flowering whereas FLOWERING LOCUS C (FLC) is a repressor (Michaels and Amasino, 1999; Sheldon et al., 2000; Hayama and Coupland, 2004; Putterill et al., 2004). Both CO and FLC regulate the expression of the floral integrator *FLOWERING LOCUS T (FT)* which acts in the apical meristem to induce floral identity genes (Abe et al., 2005; Searle et al., 2006). Mutation of *phs1-5* increased the expression of *FLC* and decreased the expression of *CO*, subsequently decreasing the expression of *FT*. These results suggest that PHS1 plays a positive role during floral transition by modulating the transcript levels of both *CO* and *FLC* (Tang et al., 2016).

MKP1 has also been shown to be involved in controlling the cell fate transition during stomata development (Tamnanloo et al., 2018). The *mkp1* mutants display significantly reduced number of stomata compared to wild type, and this reduction can be reverted by expressing MKP1 in the mutant but not by crossing with other MKPs, suggesting the major and specific role of MKP1 in regulating stomatal development (Tamnanloo et al., 2018). Further biochemical analysis shows that MKP1 acts downstream of YODA (a MAPK kinase kinase) and upstream of MPK3/MPK6; and functions at the early stage of stomatal development by promoting the differentiation of stomatal precursor cells (Tamnanloo et al., 2018).

## Dual specificity MAPK phosphatases are central hubs integrating biotic and abiotic stress

Plants are constantly exposed to diverse environmental stimuli and need to respond rapidly and effectively to these changes. MAPK phosphatases have been found biochemically and genetically to be important regulators of a broad range of stress responses, potentially functioning as central hubs for integrating multiple stress signaling pathways.

### DSP-type MAPK phosphatases are regulators of plant immune responses

**Figure 1:**
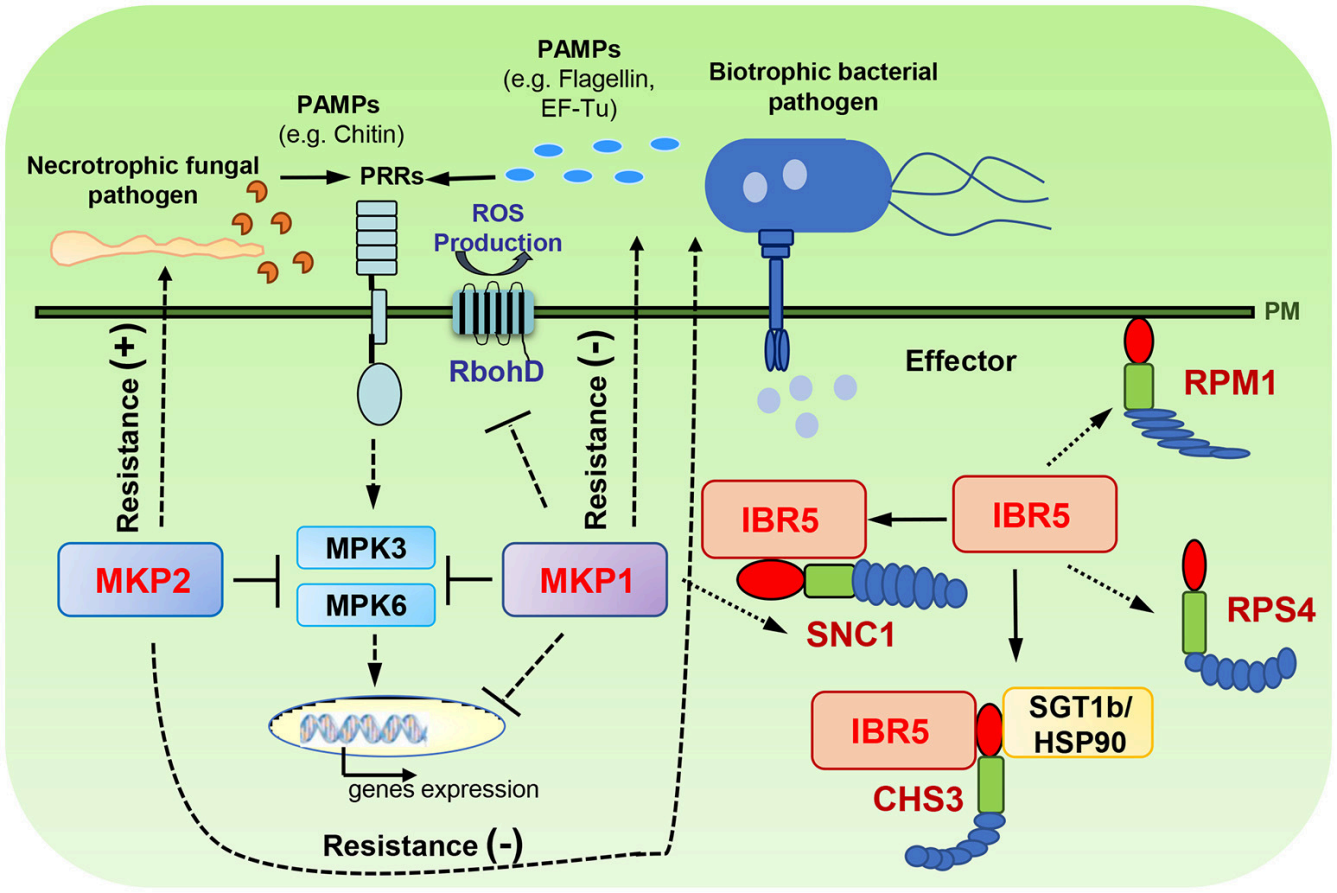
Roles of DSP-type MAPK phosphatases in modeling plant immunity. MKP1 is a negative regulator of PAMP responses (e.g., MPK3/6 activation, PAMP-induced gene expression and ROS production) and bacterial resistance. MKP1 also regulates SNC1-mediated signaling pathways. MKP2 positively regulates the resistance to necrotrophic fungal pathogen whereas negatively regulates the resistance to biotrophic bacterial pathogen. IBR5 is involved in regulating several resistance proteins, including CHS3, SNC1, RPM1, and RPS4. (−), negative regulator; (+), positive regulator. Dash lines represent the indirect regulation indicated from genetic data; and solid lines represent the direct regulation indicated from physical interactions.

In Arabidopsis, several DSP-type phosphatases have been implicated in regulating pathogen associated responses and resistance (Figure 1). MAPK PHOSPHATASE 1 (MKP1) is an important negative regulator of plant immunity. Diverse defense responses are hyper-induced in the *Arabidopsis mkp1* null mutant following pathogen-associated molecular pattern (PAMP) treatment, including activation of MPK6 and MPK3, production of reactive oxygen species (ROS), accumulation of a subset of PAMP-regulated transcripts, and inhibition of seedling growth (Anderson et al., 2011; Jiang et al., 2017b). Consistent with enhanced PAMP responses, the *mkp1* mutant also displays enhanced resistance to the virulent pathogen *Pseudomonas syringae pv. tomato (Pto)* DC3000 (hereafter referred to as DC3000) (Anderson et al., 2011). Similar to the results from *Arabidopsis*, suppression of NtMKP1 in tobacco plants also resulted in elevated resistance against multiple pathogens including a necrotrophic pathogen, *Botrytis cinerea*, and lepidopteran herbivores, *Mamestra brassica* and *Spodoptera litura* (Oka et al., 2013). Interestingly, enhanced resistance against DC3000 in Arabidopsis *mkp1* can be explained by decreased abundance of specific extracellular plant metabolites that DC3000 uses as signals to activate its virulence program (Anderson et al., 2014). Thus, MKP1 seems to regulate a novel layer of immunity against pathogen infection. However, the molecular mechanisms by which MKP1 regulates the secretion of extracellular plant metabolites need to be further explored; and how generally the regulatory roles of metabolites on pathogen resistance can be applied to other pathogen species and crop species should also be tested.

MAPK PHOSPHATASE 2 (MKP2) dephosphorylates phospho-MPK3 and phospho-MPK6 *in vitro*, and has distinct functions in regulating different pathogen interactions (Lee and Ellis, 2007; Lumbreras et al., 2010). Plants lacking MKP2 have enhanced resistance against *Ralstonia solanacearum*, a biotrophic pathogen, whereas increased susceptibility to *Botrytis cinerea*, a necrotrophic pathogen (Lumbreras et al., 2010). In addition, bimolecular fluorescence complementation (BiFC) experiments have shown that MKP2 interacts with MPK3 and MPK6 *in vivo*; and fungal elicitors decreased the MKP2-MPK3 association but increased the MKP2-MPK6 interaction (Lumbreras et al., 2010). In agreement with enhanced MKP2-MPK6 interactions, co-infiltration of MKP2 and MPK6 into *N. benthamiana* leaves significantly reduced fungal elicitor-induced HR responses compared to infiltration with MPK6 alone. Interestingly, infiltration of MPK3 did not cause significant effects in these assays (Lumbreras et al., 2010). These results suggest that MKP2 exerts differential regulation on MPK3 and MPK6 during pathogen infection.

MKPs also contribute to regulation of several resistance (R) proteins. IBR5 plays a positive role in regulating R protein CHS3, as evidenced by that mutation of *ibr5-7* suppresses the chilling-induced defense responses of *chs3-1* (Liu et al., 2015a). Biochemical studies have shown that IBR5 interacts with CHS3 through the TIR domain of CHS3 *in vivo*, and IBR5 forms a complex with chaperone protein HSP90 and SGT1b (Suppressor of the G2 allele of *skp1*) to stabilize CHS3 protein, thus increasing the accumulation of CHS3 (Liu et al., 2015a). Similarly, an *ibr5* mutant partially suppresses temperature-sensitive growth and autoimmune phenotypes resulting from constitutive activation of R protein SNC1 (Suppressor of *npr1-1*, Constitutive 1). IBR5 interacts with and promotes the accumulation of SNC1 (Liu et al., 2015a). Additionally, IBR5 is also involved in controlling disease resistance mediated by R proteins RPM1 and RPS4. The *ibr5* mutants are more susceptible to avirulent bacterial pathogens DC3000 (*avrRpm1*) and DC3000 (*avrRps4*) (Liu et al., 2015a). MKP1 has also been shown to play important roles in regulating the plant growth homeostasis by repressing inappropriate stress signaling mediated by SNC1. When the *Arabidopsis mkp1* mutation was introgressed into the Columbia ecotype from Wassilewskija, it showed weak dwarfism compared to wild type plants under standard growth conditions, and such dwarfing was caused by constitutive activation of SNC1-mediated responses (Bartels et al., 2009). These studies demonstrate the roles of MKPs in regulating plant immunity against pathogen infection through modulating multiple signaling layers in PTI and ETI.

### DSP-type MAPK phosphatases are regulators of multiple abiotic stresses

**Figure 2:**
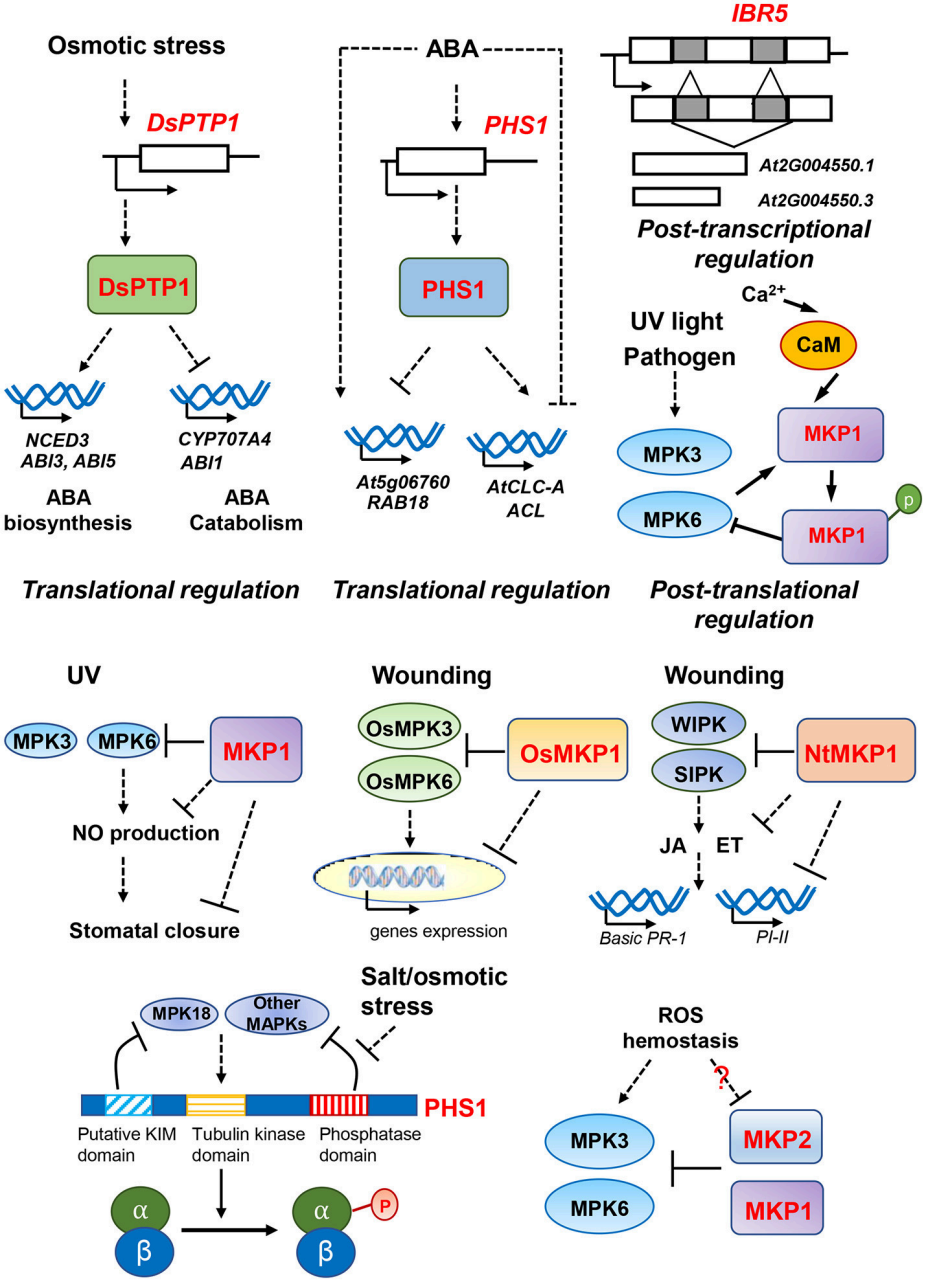
Overview of DSP-type MAPK phosphatases for multiple abiotic signaling pathways and putative regulatory mechanisms. MAPK phosphatases participate in responses to UV light, wounding, osmotic stress, salt stress and reactive oxygen species (ROS) stress. The different pathways and putative regulatory mechanisms are described in text. Dash lines represent the indirect regulation indicated from genetic data; and solid lines represent the direct regulation indicated from physical interactions.

In addition to regulating resistance against pathogen infection, MKPs also constitute important components regulating multiple abiotic stresses, including genotoxic stress, osmotic/drought stress and salinity stresses (Figure 2). For instance, the absence of MKP1 in the Arabidopsis *mkp1* mutant results in hypersensitivity to various genotoxic stresses (e.g., UV-C and methyl methanesulphonate treatments), suggesting that MKP1 plays essential roles in genotoxic stress relief (Ulm et al., 2001); and this regulation appears to be primarily through inactivation of its strongest interacting partner, MPK6, *in planta* (Ulm et al., 2002). More recently, it was found that MKP1 negatively regulates the UV-B induced stomatal closure whereas MPK6 positively regulates this process. Both aspects of regulation involve modulating hydrogen peroxide (H_2_O_2_)-induced nitric oxide (NO) production in guard cells (Li et al., 2017).

In contrast to the role of MKP1 as a positive regulator of genotoxic stress survival, MKP1 has been identified to be a negative regulator of salinity resistance as demonstrated by the fact that loss of *MKP1* increased resistance to salt stress (Ulm et al., 2002). Interestingly, ectopic over-expression of the wheat ortholog TMKP1 in Arabidopsis *mkp1* results in enhanced salt stress tolerance, indicating a positive role of TMKP1 in regulating salt stress responses and/or a possible dominant effect from ectopic overexpression. The elevated salinity resistance was accompanied by increased antioxidant enzyme activities and lower malondialdehyde (MDA), superoxide anion O^2−^ and hydrogen peroxide levels in the TMKP1 transgenic seedlings (Zaidi et al., 2016). Despite of their significant homology (49% amino acid identity), MKP1 and TMKP1 seem to act in an antagonistic manner to regulate salt stress responses, which might be explained by distinct subcellular localization and differential catalytic regulation by Ca^2+^ (Lee et al., 2008; Bartels et al., 2009; Zaïdi et al., 2010; Ghorbel et al., 2015).

During tissue wounding, MKPs have been reported to be general negative regulators in several species. In rice, the transcriptional level of OsMKP1 was rapidly induced by wounding, and knocking out OsMKP1 confers hyper-activation of two stress responsive MAPKs, OsMPK3 and OsMPK6, before and after wounding treatments (Katou et al., 2007). In addition, transcriptome analyses in *osmkp1* showed that five out of 13 genes upregulated in the mutant are clearly linked to wounding responses, suggesting that wounding responses are constitutively activated in *osmkp1* mutant (Katou et al., 2007). Similarly, tobacco NtMKP1 also negatively regulated wounding responses as NtMKP1-suppressed plants exhibited hyper-activation of wound-induced MAPKs, WIPK and SIPK (Oka et al., 2013). Furthermore, suppression of NtMKP1 increased the production of JA and ET upon wounding, and the expression of JA- or ET-inducible genes basic *PR-1* and *PI-II* were also significantly enhanced in response to wounding in the transgenic plants (Oka et al., 2013). These results suggest that NtMKP1 negatively regulates activation of MAPKs WIPK and SIPK, suppressing the accumulation of JA and ET as well as JA- or ET- inducible gene expression during wounding responses.

There is also increasing evidence highlighting the importance of MKPs in osmotic stress signaling pathways. DsPTP1 functions as a negative regulator in osmotic stress signaling in Arabidopsis seed germination and seedling establishment (Liu et al., 2015b). The null mutant *dsptp1* displayed less sensitivity to osmotic stress as shown by a higher seed germination rate and longer root length in response to osmotic stress, along with increased proline accumulation, reduced MDA content and ion leakage, and enhanced antioxidant enzyme activity (Liu et al., 2015b). Interestingly, DsPTP1 positively regulates ABA accumulation and ABA signaling in response to osmotic stress (Liu et al., 2015b). Studies show that loss of *DsPTP1* decreased ABA accumulation in *dsptp1* mutants compared to wild type plants possibly by reducing the expression of ABA-biosynthesis gene *NCED3* and increasing the expression of ABA-catabolism gene *CYP707A4* under osmotic stress condition (Liu et al., 2015b). Consistently, down regulation of DsPTP1 also suppressed the expression of positive regulators of ABA signaling such as ABI3 and ABI5 while enhancing the expression of negative regulator ABI1 (Liu et al., 2015b). In contrast to DsPTP1, PHS1 was identified to be a negative regulator of ABA signaling. ABA treatment increased transcript levels of *PHS1* gene, and *phs1-3* mutants exhibited a hypersensitivity to ABA in seed germination, light-induced stomata opening and gene expression during early development. Furthermore, knock-down mutations of PHS1 also altered the basal expression of ABA-regulated genes, enhancing the upregulation of two ABA-induced genes (*At5g06760, RAB18*) and downregulation of two ABA-repressed genes (*AtCLC-A, ACL*) (Quettier et al., 2006). However, ABA accumulation is not significantly modified in seeds and seedlings of *phs1-3* mutants, suggesting that PHS1 is involved in regulating the ABA signaling but not ABA metabolism (Quettier et al., 2006). In addition to altering ABA signaling, osmotic stress also triggers transient depolymerization of cortical microtubules in cells (Shoji et al., 2006; Wang et al., 2007; Ban et al., 2013), and PHS1 has been shown to be associated with salt/osmotic stress induced cortical microtubule depolymerization (Fujita et al., 2013). Tubulin kinase activity was suppressed by the phosphatase activity of PHS1 under normal growth conditions; but upon osmotic stress, such suppression was relieved, leading to the phosphorylation on Thr349 residue of α-tubulin, contributing to the formation of polymerization-inefficient tubulins (Fujita et al., 2013).

Studies in crops have also highlighted the importance of MKPs in osmotic stress. Rice OsIBR5 was shown to be a negative regulator of osmotic stress, because overexpression of rice OsIBR5 in tobacco plants resulted in hypersensitivity to drought and H_2_O_2_ treatments. These results might be explained by the fact that drought-induced stomatal closure was significantly reduced by overexpression of OsIBR5 in tobacco plants. In addition, OsIBR5 interacted with tobacco MAPKs SIPK and WIPK; and drought-induced activity of WIPK was compromised in OsIBR5-overexpressing tobacco plants, suggesting that OsIBR5 may regulate osmotic stress signaling through controlling drought-induced MAPKs activities (Li et al., 2012). The scenario is a bit more complicated in wheat with TMKP1 being induced in sensitive wheat varieties and repressed in tolerant ones under salt and osmotic stress, suggesting that TMKP1 may function as a negative regulator of salt and osmotic stress in the sensitive variety whereas suppression of its expression in the tolerant variety contributes to an improved stress tolerance (Zaïdi et al., 2010).

Reactive oxygen species (ROS) are generated from partial reduction of oxygen (O_2_), including hydrogen peroxide (H_2_O_2_), hydroxyl radical (HO^.^), singlet oxygen (^1^O_2_) and superoxide anion (${\text{O}}_{2}^{.-}$) (Foyer and Noctor, 2009). For plants, ROS is formed both internally and externally from cellular respiration and photosynthesis or by environmental challenges such as UV and ozone (Boldt and Scandalios, 1997; Pellinen et al., 1999; Mittler, 2002). To survive, plants need to balance redox homeostasis because high levels of ROS accumulation is detrimental to plant cells (Moller and Jensen, 2007). Several studies have suggested that MKPs are involved in ROS signaling and responses. Silencing of MKP2 in plants resulted in hypersensitivity to ozone treatments and prolonged activation of MPK3 and MPK6. The seedling growth of *mkp2* mutants was more severely inhibited by the oxidative agent methyl-viologen (MV) compared to wild type plants (Lee and Ellis, 2007; Lumbreras et al., 2010). However, the accumulation of superoxide radical anions and hydrogen peroxide was not significantly altered in the *mkp2* mutants (Lumbreras et al., 2010). These results suggest that loss of MKP2 disrupts oxidative stress responses, but not through changing the ROS levels and homeostasis but by enhancing the susceptibility of *mkp2* plants to ROS accumulation. MKP1 is also implicated to play a role in ROS signaling. When treated with bacterial PAMP elf26 (26 conserved amino acids from bacterial elongation factor EF-Tu), *mkp1* mutants produced more ROS than in wild type plants (Anderson et al., 2011). Furthermore, a sequence of peroxisomal targeting signal 1 (PTS1) was identified in the MKP1 protein; and the full-length MKP1 protein was observed to transport from cytoplasm to peroxisomes in response to different biotic and abiotic stresses when transiently expressed in mesophyll protoplasts (Kataya et al., 2015). However, a detailed mechanism of how MKP1 or MKP2 regulates the ROS signaling remains unclear.

## Regulatory mechanisms of dual specificity MAPK phosphatases

Plants have multiple perception systems to sense distinct environmental signals. However, multiple signaling pathways appear to merge together downstream and share common modules for transducing and amplifying signals. Such mechanisms are very efficient and effective for plants to quickly respond to the environment. However, questions arise about how specificity of signaling outputs is achieved if different signaling networks all coalesce in shared hubs. One possibility is that MKPs, which participate in diverse signaling pathways, may play a key role in integrating these signaling pathways. Therefore, precise regulation of MKPs is likely critical for plants to generate the correct biological responses. Recent progress on the regulatory mechanisms of MKPs may facilitate our understanding of the role of MKPs in mediating crosstalk between different signaling pathways and ensuring the signal specificity.

### Transcriptional and post-transcriptional regulation of MKPs

Transcriptional and post-transcriptional regulation provide potential layers of control, and the expression levels of MKPs have been found to respond differentially to various stress signals (Figure 2). For instance, transcript levels of *PHS1* are induced by ABA treatment (Quettier et al., 2006). Similarly, rice *OsIBR5*'s transcription is also enhanced by ABA treatment, other osmotic stresses including PEG6000, and hydrogen peroxide (H_2_O_2_) treatments (Li et al., 2012); and rice *OsMKP1* and tobacco *NtMKP1* transcripts increase after wounding (Yamakawa et al., 2004; Katou et al., 2007). Interestingly, different expression patterns of wheat *TMKP1* were observed between different wheat cultivars with induced expression in sensitive cultivars and repressed in tolerant ones (Zaïdi et al., 2010). In contrast, the expression of Arabidopsis *MKP1* does not appear to be significantly altered after exposure to either UV-B treatment or pathogen infection (González Besteiro and Ulm, 2013; Jiang et al., 2017a), suggesting that *MKP1* is mainly under post-translational regulation. Although the regulation of the *IBR5*'s transcription has not been reported, it has been shown that *IBR5* is post-transcriptionally regulated, generating two transcripts, *AT2G004550.1* and *AT2G04550.3*, by alternative splicing to produce two IBR5 isoforms IBR5.1 and IBR5.3 (Jayaweera et al., 2014). IBR5.1 and IBR5.3 isoforms have overlapping, but also distinct, functions as the mutant alleles share many phenotypes but also confer distinct morphological defects (Jayaweera et al., 2014). Part of these differences may be explained by the fact that IBR5 isoforms have displayed different localization patterns, with IBR5.1 localized in both the nucleus and cytoplasm whereas IBR5.3 is exclusively in nucleus (Jayaweera et al., 2014).

### Post-translational protein modification alters phosphatase activity of MKPs

Modulating the activity of MKPs can change the activation profiles of their target kinases (e.g., MAPKs), contributing to differences in amplitude and duration of signaling. Many examples have shown that the phosphatase activity of MKPs can be regulated by post-translational modification. Mammalian MKP1s (not an orthologue of plant MKP1s) and yeast Msg5 are phosphorylated by their own substrates MAPKs, and phosphorylation regulates not only the activities but also the abundance of the phosphatases, establishing an efficient negative feedback loop (Brondello et al., 1999; Sohaskey and Ferrell, 2002; Li et al., 2007). For instance, in hamster fibroblasts, the active p42/44 MAPKs phosphorylate MKP1, leading to the reduction of proteasomal degradation, thereby, stabilizing the MKP1 protein (Brondello et al., 1999). Similar regulatory mechanisms have been demonstrated in plants. In Arabidopsis, MKP1 is phosphorylated by MPK6 *in vitro*; and phosphorylation increased its phosphatase activity (Park et al., 2011). Similarly, the phosphatase activity of MKP2 is elevated in the presence of either recombinant MPK3 or MPK6 *in vitro* (Lee and Ellis, 2007). This level of regulation has also been found in crop species. For instance, the phosphatase activity of tobacco NtMKP1 is also increased by co-incubation of its substrates such as SIPK (Katou et al., 2005), and the catalytic activity of wheat TMKP1 is significantly enhanced by co-incubation of TMPK3 (Zaïdi et al., 2010). In addition to *in vitro* studies, *in vivo* studies using Arabidopsis transgenic lines expressing MKP1 protein have also shown that MKP1 is phosphorylated in response to both UV-B treatment and pathogen challenges (González Besteiro and Ulm, 2013; Jiang et al., 2017a). The phosphorylation of MKP1 contributes to its stability and is required for its biological function during both UV-B stresses and pathogen infection (González Besteiro and Ulm, 2013; Jiang et al., 2017a).

Another important role of phosphorylation is serving as a docking site for recruiting interacting proteins. For wheat TMKP1, it was shown that TMKP1 associates with 14-3-3 proteins through a canonical model 14-3-3 binding motif (^574^KLPSLP^579^); and phosphorylation of TMKP1 is required for the interaction (Ghorbel et al., 2017). In addition, interaction with 14-3-3 proteins increased the phosphatase activity of TMKP1, and TMKP1 activation was further enhanced by Mn^2+^ (Ghorbel et al., 2017). Thus, MKPs phosphorylation can modify phosphatase activity, protein stability, and interaction with other proteins, all of which may facilitate roles of MKPs in regulating different responses and also possibly integrating distinct signals.

### Differential localization of MKPs and interacting MAPKs may affect specific signaling regulation

Differential localization of MKPs could lead to regulation of subcellular pools of MAPKs with different positional information. More specifically, where the kinases are located when active may be important in conveying specific signals. For instance, expression of PHS1-GFP fusion protein in tobacco cells and epidermal cells of Arabidopsis showed that PHS1 was mainly localized in the cytoplasm (Walia et al., 2009; Pytela et al., 2010). Accordingly, the interaction between PHS1 and MKP18 also occurred in cytoplasm (Walia et al., 2009). The cytoplasmic localization of PHS1 may also explain the predominant role of PHS1 in regulating microtubule organization through controlling the phosphorylation status of tubulins. The localization of Arabidopsis IBR5 is less clear. Studies using transgenic plants expressing fluorescently tagged IBR5 and its interacting MAPK MPK12 under constitutive promoters indicated that both IBR5 and MPK12 were primarily localized in the nucleus (Lee et al., 2009). However, transgenic plants with IBR5 driven by its native promoter indicate that IBR5 is distributed in both the nucleus and cytoplasm (Jayaweera et al., 2014). Arabidopsis MKP1 was found to be predominantly in cytoplasm, and its interaction with MPK6 was also mainly cytoplasmic (Bartels et al., 2009). Later studies report that MKP1 harbors a non-canonical PTS1-like tripeptide which is conserved in MKP1 orthologs (Kataya et al., 2015). Experiments with transient expression of MKP1 in mesophyll protoplasts indicate that MKP1 can move from cytoplasm into peroxisomes under stressful conditions, suggesting a potential role for MKP1 in regulating peroxisomal functions by reversible phosphorylation (Kataya et al., 2015). Also interacting with MPK3/6, Arabidopsis MKP2 was found primarily localized in the nucleus of MKP2-YFP-expressing plants, the same subcellular compartment where ozone-activated MAPKs were translocated (Ahlfors et al., 2004; Lee and Ellis, 2007). However, transient expression of GFP-tagged MKP2 in tobacco cells showed MKP2 localized in both nucleus and cytoplasm, and BiFC studies demonstrated that the interaction between MKP2 and MPK3/6 occurred also in both the nucleus and the cytoplasm (Lumbreras et al., 2010). Therefore, even sharing the same target MAPKs, MKP1, and MKP2 likely play different roles in regulating pathogen infections and other responses, which may result from differential localization of MKP1 and MKP2 as well as differences in the compartments where they interact with MAPKs. In addition, expression of MKPs may also affect the shuttling of MAPKs between nucleus and the cytoplasm. As shown by expression in BY2 tobacco cells, a GFP-TMKP1 fusion protein accumulated in the nucleus with TMPK3-RFP or TMPK6-RFP also accumulating in the nucleus (Zaïdi et al., 2010). However, when expressing the truncated form GFP-TMKP1ΔN_1−133_ (truncating the N-terminal non-catalytic region), which was mainly localized in cytoplasm, the localization of both MAPKs also changed to the cytoplasm (Zaïdi et al., 2010). One of the possible explanations is that MKPs alter the phosphorylation status of MAPKs which plays essential roles in partitioning between the nucleus and cytoplasm, a mechanism that is well established in yeast and mammalian system (Lenormand et al., 1993; Ferrigno et al., 1998; Gatis et al., 1998; Khokhlatchev et al., 1998). Another possibility is that MKPs serve as a nuclear and/or cytoplasmic tether for these MAPKs as has been shown in yeast (Mattison and Ota, 2000). Together, MKPs localized in differential subcellular compartments may contribute to controlling different pools of active MAPKs as well as the shuttling of MAPKs between the nucleus and the cytoplasm, leading to proper responses to specific signal inputs.

### MKPs may play a role in integrating Ca^2+^ signaling pathways

Phosphatases may also contribute to signal specificity through combinatorial integration with other kinases. MAPK phosphatases in multiple species have been reported to be calmodulin (CaM) binding proteins, where CaM is a Ca^2+^-sensor protein, indicating a potential link to calcium-mediated signaling pathways. For instance, tobacco NtMKP1 and rice OsMKP1 bind CaM through a single putative CaM binding domain (CaMBD) (Yamakawa et al., 2004; Katou et al., 2007), and Arabidopsis MKP1 and DsPTP1 bind CaM via two different CaMBDs (Yoo et al., 2004; Lee et al., 2008). Therefore, these interaction between CaM and MKPs may contribute to the specific regulation of MKPs. For instance, *in vitro* studies found that binding of CaM increases phosphatase activities on p-nitrophenyl phosphate (pNPP) substrates but decreases phosphatase activity on the phosphotyrosine of myelin basic protein (MBP), suggesting that calmodulin differentially regulates substrate specificity (Yoo et al., 2004). In addition, different CaMs have different binding affinities to MKPs. Bacterially expressed tobacco NtMKP1 physically interacts with three plant-specific types of CaMs, showing high affinity to NtCaM1 and NtCaM3 but lower affinity to NtCaM13 (Yamakawa et al., 2004). Even the same CaM has different binding affinities to different CaMBDs in MKPs. For instance, two CaMBDs (CaMBDI and CaMBDII) are present in the Arabidopsis MKP1, and CaM binds to both CaMBDs in a Ca^2+^-dependent manner (Lee et al., 2008). The binding affinity of CaMDBII was found to be higher than that of CaMBDI; and mutations on W453, L456 in CaMBDI and W678, I684 in CaMBDII disrupt the binding (Lee et al., 2008). Binding of CaMs to these two CaMBDs increased MKP1 phosphatase activity about 2-fold (Lee et al., 2008), indicating the possible regulation of MAPK phosphatases through Ca^2+^ signaling pathways. Interestingly, the CaMBDI mutant (W453R) but not CaMBDII mutant (W678R) can be activated by CaM, which indicates that CaMBDII plays a more important role than CaMBDI for the regulation of MKP1 (Lee et al., 2008). There may be additional variation between species, however, as wheat TMKP1 binds to CaM in a Ca^2+^-dependent manner as in Arabidopsis, but binding of CaM inhibits the phosphatase activity of TMKP1. Interestingly, the presence of Mn^2+^ can reverse the inhibitory effect of CaM binding, resulting in enhanced phosphatase activity of TMKP1 (Ghorbel et al., 2015). Together, these results indicate that regulation of MKPs may provide a connection between Ca^2+^- and MAPK signaling. Such regulation may contribute to the differential activation of MKPs, eventually leading to modulation of different pathways to produce biologically specific responses.

## Conclusions and future prospects

This review summarizes current knowledge of the roles of MKPs in multiple signaling pathways. MKPs are involved in many aspects of a plant's life cycle, including growth and development as well as the adaption to various biotic and abiotic stresses. As different signaling pathways integrate through MKPs, it is critical for MKPs to undergo precise regulation to generate correct biological outcomes. These investigations of regulatory mechanisms have provided insights into putative mechanisms that might explain how the phosphatases assist in producing specific signals. The potential combinatorial complexity of different transcriptional/post-transcriptional regulations, differential localization affecting subcellular pools of MAPKs, and the possibility to integrate information from other pathways may have profound effects on organizing the amplitude and duration of targeted kinases.

As important as the MKPs appear to be, most of our knowledge regarding their functions is limited to their roles in negatively regulating MAPK signaling, and few other potential upstream and/or downstream targets have yet to be characterized. Genome-wide analyses will certainly facilitate a better understanding of MKPs-mediated signaling pathways by identification of both molecular players in the pathways as well as additional putative MKP targets. A better understanding of how plants coordinate and balance different signaling pathways in response to diverse environmental stimuli could lead to more rationally designed strategies for improving crop yield under changing environmental conditions. Promising results such as the deletion of MKP1 resulting in enhanced resistance to various biotic and abiotic stresses without compromising plant growth (Ulm et al., 2001, 2002; Anderson et al., 2011, 2014) suggest that it may be possible to produce crops with elevated resistance against adverse environmental stresses. As apparent integrators of diverse signaling pathways, MKPs are important targets for modulating cross-talk to help overcome barriers for the improvement of plant resistance.

## Author contributions

LJ and SP conceived the main subject of this review and wrote the manuscript. YC and LL provided the suggestions and revisions.

### Conflict of interest statement

The authors declare that the research was conducted in the absence of any commercial or financial relationships that could be construed as a potential conflict of interest.

## References

[B1] Abe M. Kobayashi Y. Yamamoto S. Daimon Y. Yamaguchi A. Ikeda Y.. (2005). FD, a bZIP protein mediating signals from the floral pathway integrator FT at the shoot apex. Science 309, 1052–1056. 10.1126/science.1115983 16099979

[B2] Ahlfors R. Macioszek V. Rudd J. Brosche M. Schlichting R. Scheel D.. (2004). Stress hormone-independent activation and nuclear translocation of mitogen-activated protein kinases in *Arabidopsis thaliana* during ozone exposure. Plant J. 40, 512–522. 10.1111/j.1365-313X.2004.02229.x 15500467

[B3] Alessi D. R. Smythe C. Keyse S. M. (1993). The human CL100 gene encodes a Tyr/Thr-protein phosphatase which potently and specifically inactivates MAP kinase and suppresses its activation by oncogenic ras in Xenopus oocyte extracts. Oncogene 8, 2015–2020. 8390041

[B4] Anderson J. C. Bartels S. González Besteiro M. A. Shahollari B. Ulm R. Peck S. C. (2011). Arabidopsis MAP Kinase Phosphatase 1 (AtMKP1) negatively regulates MPK6-mediated PAMP responses and resistance against bacteria. Plant J. 67, 258–268. 10.1111/j.1365-313X.2011.04588.x 21447069

[B5] Anderson J. C. Wan Y. Kim Y. M. Pasa-Tolic L. Metz T. O. Peck S. C. (2014). Decreased abundance of type III secretion system-inducing signals in Arabidopsis mkp1 enhances resistance against *Pseudomonas syringae*. Proc. Natl. Acad. Sci. U.S.A. 111, 6846–6851. 10.1073/pnas.1403248111 24753604 PMC4020108

[B6] Ban Y. Kobayashi Y. Hara T. Hamada T. Hashimoto T. Takeda S.. (2013). ?-tubulin is rapidly phosphorylated in response to hyperosmotic stress in rice and Arabidopsis. Plant Cell Physiol. 54, 848–858. 10.1093/pcp/pct065 23628996

[B7] Bartels S. Anderson J. C. González Besteiro M. A. Carreri A. Hirt H. Buchala A.. (2009). MAP KINASE PHOSPHATASE1 and PROTEIN TYROSINE PHOSPHATASE1 are repressors of salicylic acid synthesis and SNC1-mediated responses in Arabidopsis. Plant Cell 21, 2884–2897. 10.1105/tpc.109.067678 19789277 PMC2768924

[B8] Bartels S. González Besteiro M. A. Lang D. Ulm R. (2010). Emerging functions for plant MAP kinase phosphatases. Trends Plant Sci. 15, 322–329. 10.1016/j.tplants.2010.04.003 20452268

[B9] Boldt R. Scandalios J. G. (1997). Influence of UV-light on the expression of the Cat2 and Cat3 catalase genes in maize. Free Radic. Biol. Med. 23, 505–514. 10.1016/S0891-5849(97)00111-1 9214589

[B10] Brock A. K. Willmann R. Kolb D. Grefen L. Lajunen H. M. Bethke G.. (2010). The Arabidopsis mitogen-activated protein kinase phosphatase PP2C5 affects seed germination, stomatal aperture, and abscisic acid-inducible gene expression. Plant Physiol. 153, 1098–1111. 10.1104/pp.110.156109 20488890 PMC2899920

[B11] Brondello J. M. Pouysségur J. McKenzie F. R. (1999). Reduced MAP kinase phosphatase-1 degradation after p42/p44MAPK-dependent phosphorylation. Science 286, 2514–2517. 10.1126/science.286.5449.2514 10617468

[B12] Colcombet J. Hirt H. (2008). Arabidopsis MAPKs: a complex signalling network involved in multiple biological processes. Biochem. J. 413, 217–226. 10.1042/BJ20080625 18570633

[B13] Ferrigno P. Posas F. Koepp O. Saito H. Silver P. A. (1998). Regulated nucleo/cytoplasmic exchange of HOG1 MAPK requires the importin beta homologs NMO5 and XPO1. EMBO J. 17, 5606–5614. 10.1093/emboj/17.19.5606 9755161 PMC1170889

[B14] Foyer C. H. Noctor G. (2009). Redox regulation in photosynthetic organisms: signaling, acclimation, and practical implications. Antioxid. Redox. Signal 11, 861–905. 10.1089/ars.2008.2177 19239350

[B15] Fuchs S. Grill E. Meskiene I. Schweighofer A. (2012). Type 2C protein phosphatases in plants. FEBS J. 280, 681–693. 10.1111/j.1742-4658.2012.08670.x 22726910

[B16] Fujita S. Pytela J. Hotta T. Kato T. Hamada T. Akamatsu R.. (2013). An atypical tubulin kinase mediates stress-induced microtubule depolymerization in Arabidopsis. Curr. Biol. 23, 1969–1978. 10.1016/j.cub.2013.08.006 24120637

[B17] Galletti R. Ferrari S. De Lorenzo G. (2011). Arabidopsis MPK3 and MPK6 play different roles in basal and oligogalacturonide- or flagellin-induced resistance against Botrytis cinerea. Plant Physiol. 157, 804–814. 10.1104/pp.111.174003 21803860 PMC3192574

[B18] Gatis F. Degols G. Shiozaki K. Russell P. (1998). Phosphorylation and association with the transcription factor Atf1 regulate localization of Spc1/Sty1 stress-activated kinase in fission yeast. Genes Dev. 12, 1464–1473. 10.1101/gad.12.10.1464 9585506 PMC316836

[B19] Ghorbel M. Cotelle V. Ebel C. Zaidi I. Ormancey M. Galaud J. P.. (2017). Regulation of the wheat MAP kinase phosphatase 1 by 14-3-3 proteins. Plant Sci. 257, 37–47. 10.1016/j.plantsci.2017.01.006 28224917

[B20] Ghorbel M. Zaidi I. Robe E. Ranty B. Mazars C. Galaud J. P.. (2015). The activity of the wheat MAP kinase phosphatase 1 is regulated by manganese and by calmodulin. Biochimie 108, 13–19. 10.1016/j.biochi.2014.10.021 25447143

[B21] González Besteiro M. A. Ulm R. (2013). Phosphorylation and stabilization of Arabidopsis MAP kinase phosphatase 1 in response to UV-B stress. J. Biol. Chem. 288, 480–486. 10.1074/jbc.M112.434654 23188831 PMC3537045

[B22] Gray W. M. Kepinski S. Rouse D. Leyser O. Estelle M. (2001). Auxin regulates SCF (TIR1)-dependent degradation of Aux/IAA proteins. Nature 414, 271–276. 10.1038/35104500 11713520

[B23] Gupta R. Huang Y. Kieber J. Luan S. (1998). Identification of a dual-specificity protein phosphatase that inactivates a MAP kinase from Arabidopsis. Plant J. 16, 581–589. 10.1046/j.1365-313x.1998.00327.x 10036776

[B24] Hamel L. P. Nicole M. C. Sritubtim S. Morency M. J. Ellis M. Ehlting J.. (2006). Ancient signals: comparative genomics of plant MAPK and MAPKK gene families. Trends Plant Sci. 11, 192–198. 10.1016/j.tplants.2006.02.007 16537113

[B25] Hayama R. Coupland G. (2004). The molecular basis of diversity in the photoperiodic flowering responses of Arabidopsis and rice. Plant Physiol. 135, 677–684. 10.1104/pp.104.042614 15208414 PMC514104

[B26] Ichimura K. Shinozaki K. Tena G. Sheen J. Henry Y. Champion A. (2002). Mitogen-activated protein kinase cascades in plants: a new nomenclature. Trends Plant Sci. 7, 301–308. 10.1016/S1360-1385(02)02302-6 12119167

[B27] Jayaweera T. Siriwardana C. Dharmasiri S. Quint M. Gray W. M. Dharmasiri N. (2014). Alternative Splicing of Arabidopsis IBR5 Pre-mRNA Generates Two IBR5 Isoforms with Distinct and Overlapping Functions. PLoS ONE 9:e102301. 10.1371/journal.pone.0102301 25144378 PMC4140696

[B28] Jiang L. Anderson J. C. Gonzalez Besteiro M. A. Peck S. C. (2017a). Phosphorylation of Arabidopsis MAP Kinase Phosphatase 1 (MKP1) is required for PAMP responses and resistance against Bacteria. Plant Physiol. 175, 1839–1852. 10.1104/pp.17.01152 29070514 PMC5717735

[B29] Jiang L. Wan Y. Anderson J. C. Hou J. Islam S. M. Cheng J.. (2017b). Genetic dissection of Arabidopsis MAP kinase phosphatase 1-dependent PAMP-induced transcriptional responses. J. Exp. Bot. 68, 5207–5220. 10.1093/jxb/erx335 29045691 PMC5853853

[B30] Johnson K. L. Ramm S. Kappel C. Ward S. Leyser O. Sakamoto T. (2015). The Tinkerbell (Tink) Mutation Identifies the Dual-Specificity MAPK Phosphatase INDOLE-3-BUTYRIC ACID-RESPONSE5 (IBR5) as a novel regulator of organ size in Arabidopsis. PLoS ONE 10:e0131103 10.1371/journal.pone.0131103 26147117 PMC4492785

[B31] Kataya A. R. Schei E. Lillo C. (2015). MAP kinase phosphatase 1 harbors a novel PTS1 and is targeted to peroxisomes following stress treatments. J. Plant Physiol. 179, 12–20. 10.1016/j.jplph.2015.03.002 25817413

[B32] Katou S. Karita E. Yamakawa H. Seo S. Mitsuhara I. Kuchitsu K. (2005). Catalytic activation of the plant MAPK phosphatase NtMKP1 by its physiological substrate salicylic acid-induced protein kinase but not by calmodulins. J. Biol. Chem. 280, 39569–39581. 10.1074/jbc.M508115200 16183637

[B33] Katou S. Kuroda K. Seo S. Yanagawa Y. Tsuge T. Yamazaki M.. (2007). A calmodulin-binding mitogen-activated protein kinase phosphatase is induced by wounding and regulates the activities of stress-related mitogen-activated protein kinases in rice. Plant Cell Physiol. 48, 332–344. 10.1093/pcp/pcm007 17218330

[B34] Kerk D. Templeton G. Moorhead G. B. (2008). Evolutionary radiation pattern of novel protein phosphatases revealed by analysis of protein data from the completely sequenced genomes of humans, green algae, and higher plants. Plant Physiol. 146, 351–367. 10.1104/pp.107.111393 18156295 PMC2245839

[B35] Keyse S. M. Emslie E. A. (1992). Oxidative stress and heat shock induce a human gene encoding a protein-tyrosine phosphatase. Nature 359, 644–646. 10.1038/359644a0 1406996

[B36] Khokhlatchev A. V. Canagarajah B. Wilsbacher J. Robinson M. Atkinson M. Goldsmith E.. (1998). Phosphorylation of the MAP kinase ERK2 promotes its homodimerization and nuclear translocation. Cell 93, 605–615. 10.1016/S0092-8674(00)81189-7 9604935

[B37] Lee J. S. Ellis B. E. (2007). Arabidopsis MAPK phosphatase 2 (MKP2) positively regulates oxidative stress tolerance and inactivates the MPK3 and MPK6 MAPKs. J. Biol. Chem. 282, 25020–25029. 10.1074/jbc.M701888200 17586809

[B38] Lee J. S. Wang S. Sritubtim S. Chen J. G. Ellis B. E. (2009). Arabidopsis mitogen-activated protein kinase MPK12 interacts with the MAPK phosphatase IBR5 and regulates auxin signaling. Plant J. 57, 975–985. 10.1111/j.1365-313X.2008.03741.x 19000167

[B39] Lee K. Song E. H. Kim H. S. Yoo J. H. Han H. J. Jung M. S.. (2008). Regulation of MAPK phosphatase 1 (AtMKP1) by calmodulin in Arabidopsis. J. Biol. Chem. 283, 23581–23588. 10.1074/jbc.M801549200 18579522 PMC3259760

[B40] Lenormand P. Sardet C. Pagès G. L'Allemain G. Brunet A. Pouysségur J. (1993). Growth factors induce nuclear translocation of MAP kinase (p42mapk and p44mapk) but not of their activator MAP kinase kinase (p45mapkk) in fibroblasts. J. Cell Biol. 122, 1079–1088. 10.1083/jcb.122.5.1079 8394845 PMC2119624

[B41] Li C. Scott D. A. Hatch E. Tian X. Mansour S. L. (2007). Dusp6 (Mkp3) is a negative feedback regulator of FGF-stimulated ERK signaling during mouse development. Development 134, 167–176. 10.1242/dev.02701 17164422 PMC2424197

[B42] Li F. C. Wang J. Wu M. M. Fan C. M. Li X. He J. M. (2017). Mitogen-activated protein kinase phosphatases affect UV-B-induced stomatal closure via controlling NO in Guard Cells. Plant Physiol. 173, 760–770. 10.1104/pp.16.01656 27837091 PMC5210765

[B43] Li Y. Feng D. Zhang D. Su J. Zhang Y. Li Z. (2012). Rice MAPK phosphatase IBR5 negatively regulates drought stress tolerance in transgenic *Nicotiana tabacum*. Plant Sci. 88–189, 10–18. 10.1016/j.plantsci.2012.02.005 22525239

[B44] Liu J. Yang H. Bao F. Ao K. Zhang X. Zhang Y.. (2015a). IBR5 modulates temperature-dependent, R Protein CHS3-mediated defense responses in Arabidopsis. PLoS Genet. 11:e1005584. 10.1371/journal.pgen.1005584 26451844 PMC4599859

[B45] Liu R. Liu Y. Ye N. Zhu G. Chen M. Jia L.. (2015b). AtDsPTP1 acts as a negative regulator in osmotic stress signalling during Arabidopsis seed germination and seedling establishment. J. Exp. Bot. 66, 1339–1353. 10.1093/jxb/eru484 25540435 PMC4339596

[B46] Lumbreras V. Vilela B. Irar S. Solé M. Capellades M. Valls M.. (2010). MAPK phosphatase MKP2 mediates disease responses in Arabidopsis and functionally interacts with MPK3 and MPK6. Plant J. 63, 1017–1030. 10.1111/j.1365-313X.2010.04297.x 20626661

[B47] Mattison C. P. Ota M. (2000). Two protein tyrosine phosphatases, Ptp2 and Ptp3, modulate the subcellular localization of the Hog1 MAP kinase in yeast. Genes Dev. 14, 1229–1235. 10.1101/grad.14.10.1229 10817757 PMC316617

[B48] Meskiene I. Baudouin E. Schweighofer A. Liwosz A. Jonak C. Rodriguez P. L.. (2003). Stress-induced protein phosphatase 2C is a negative regulator of a mitogen-activated protein kinase. J. Biol. Chem. 278, 18945–18952. 10.1074/jbc.M300878200 12646559

[B49] Michaels S. D. Amasino R. M. (1999). FLOWERING LOCUS C encodes a novel MADS domain protein that acts as a repressor of flowering. Plant Cell 11, 949–956. 10.1105/tpc.11.5.949 10330478 PMC144226

[B50] Mine A. Berens M. L. Nobori T. Anver S. Fukumoto K. Winkelmüller T. M.. (2017). Pathogen exploitation of an abscisic acid- and jasmonate-inducible MAPK phosphatase and its interception by Arabidopsis immunity. Proc. Natl. Acad. Sci. U.S.A. 114, 7456–7461. 10.1073/pnas.1702613114 28652328 PMC5514735

[B51] Mittler R. (2002). Oxidative stress, antioxidants and stress tolerance. Trends Plant Sci. 7, 405–410. 10.1016/S1360-1385(02)02312-9 12234732

[B52] Moller I. M. Jensen P. E. (2007). A. Hansson, Oxidative modifications to cellular components in plants. Annu. Rev. Plant Biol. 58, 459–481. 10.1146/annurev.arplant.58.032806.103946 17288534

[B53] Monroe-Augustus M. Zolman B. K. Bartel B. (2003). IBR5, a dual-specificity phosphatase-like protein modulating auxin and abscisic acid responsiveness in Arabidopsis. Plant Cell 15, 2979–2991. 10.1105/tpc.017046 14630970 PMC282844

[B54] Naoi K. Hashimoto T. (2004). A semidominant mutation in an Arabidopsis mitogen-activated protein kinase phosphatase-like gene compromises cortical microtubule organization. Plant Cell 16, 1841–1853. 10.1105/tpc.021865 15208393 PMC514165

[B55] Oka K. Amano Y. Katou S. Seo S. Kawazu K. Mochizuki A.. (2013). Tobacco MAP kinase phosphatase (NtMKP1) negatively regulates wound response and induced resistance against necrotrophic pathogens and lepidopteran herbivores. Mol. Plant Microbe Interact. 26, 668–675. 10.1094/MPMI-11-12-0272-R 23425101

[B56] Park H. C. Song E. H. Nguyen X. C. Lee K. Kim K. E. Kim H. S.. (2011). Arabidopsis MAP kinase phosphatase 1 is phosphorylated and activated by its substrate AtMPK6. Plant Cell Rep. 30, 1523–1531. 10.1007/s00299-011-1064-4 21455789

[B57] Pellinen R. Palva T. Kangasjarvi J. (1999). Short communication: subcellular localization of ozone-induced hydrogen peroxide production in birch (*Betula pendula*) leaf cells. Plant J. 20, 349–356. 10.1046/j.1365-313X.1999.00613.x 10571895

[B58] Putterill J. Laurie R. Macknight R. (2004). It's time to flower: the genetic control of flowering time. Bio. Essays 26, 363–373. 10.1002/bies.20021 15057934

[B59] Pytela J. Kato T. Hashimoto T. (2010). Mitogen-activated protein kinase phosphatase PHS1 is retained in the cytoplasm by nuclear extrusion signal-dependent and independent mechanisms. Planta 231, 1311–1322. 10.1007/s00425-010-1135-8 20224945

[B60] Quettier A. L. Bertrand C. Habricot V. Miginiac E. Agnes C. Jeannette E. (2006). The phs1-3 mutation in a putative dual-specificity protein tyrosine phosphatase gene provides hypersensitive responses to abscisic acid in *Arabidopsis thaliana*. Plant J. 47, 711–719. 10.1111/j.1365-313X.2006.02823.x 16889651

[B61] Schweighofer A. Kazanaviciute V. Scheik E. Teige M. Doczi R. Hirt H.. (2007). The PP2C-type phosphatase AP2C1, which negatively regulates MPK4 and MPK6, modulates innate immunity, jasmonic acid, and ethylene levels in Arabidopsis. Plant Cell 19, 2213–2224. 10.1105/tpc.106.049585 17630279 PMC1955703

[B62] Searle I. He Y. Turck F. Vincent C. Fornara F. Krob¨er S.. (2006). The transcription factor FLC confers a flowering response to vernalization by repressing meristem competence and systemic signaling in Arabidopsis. Genes Dev. 20, 898–912. 10.1101/gad.373506 16600915 PMC1472290

[B63] Shankar A. Agrawal N. Sharma M. Pandey A. Girdhar K. Pandey M. (2015). Role of Protein Tyrosine Phosphatases in Plants. Curr. Genomics. 16, 224–236. 10.2174/1389202916666150424234300 26962298 PMC4765517

[B64] Sheldon C. C. Rouse D. T. Finnegan E. J. Peacock W. J. Dennis E. S. (2000). The molecular basis of vernalization: the central role of FLOWERING LOCUS C (FLC). Proc. Natl. Acad. Sci. U.S.A. 97, 3753–3758. 10.1073/pnas.97.7.3753 10716723 PMC16312

[B65] Shi Y. (2009). Serine/Threonine phosphatases: mechanism through structure. Cell 139, 468–484. 10.1016/j.cell.2009.10.006 19879837

[B66] Shoji T. Suzuki K. Abe T. Kaneko Y. Shi H. Zhu J. K.. (2006). Salt stress affects cortical microtubule organization and helical growth in Arabidopsis. Plant Cell Physiol. 47, 1158–1168. 10.1093/pcp/pcj090 16861712

[B67] Sidonskaya E. Schweighofer A. Shubchynskyy V. Kammerhofer N. Hofmann J. Wieczorek K.. (2016). Plant resistance against the parasitic nematode Heterodera schachtii is mediated by MPK3 and MPK6 kinases, which are controlled by the MAPK phosphatase AP2C1 in Arabidopsis. J. Exp. Bot. 67, 107–118. 10.1093/jxb/erv440 26438412 PMC4682428

[B68] Singh A. Pandey A. Srivastava A. K. Tran L. S. Pandey G. K. (2016). Plant protein phosphatases 2C: from genomic diversity to functional multiplicity and importance in stress management. Crit. Rev. Biotechnol. 36:1023–1035. 10.3109/07388551.2015.1083941 26380928

[B69] Sohaskey M. L. Ferrell J. E. Jr. (2002). Activation of p42 mitogen activated protein kinase (MAPK), but not c-Jun NH2-terminal kinase, induces phosphorylation and stabilization of MAPK phosphatase XCL100 in Xenopus oocytes. Mol. Biol. Cell 13, 454–468. 10.1091/mbc.01-11-0553 11854404 PMC65641

[B70] Strader L. C. Bartel B. (2009). The Arabidopsis PLEIOTROPIC DRUG RESISTANCE8/ABCG36 ATP binding cassette transporter modulates sensitivity to the auxin precursor indole-3-butyric acid. Plant Cell 21, 1992–2007. 10.1105/tpc.109.065821 19648296 PMC2729616

[B71] Strader L. C. Monroe-Augustus M. Bartel B. (2008a). The IBR5 phosphatase promotes Arabidopsis auxin responses through a novel mechanism distinct from TIR1-mediated repressor degradation. BMC Plant Biol. 8:41. 10.1186/1471-2229-8-41 18423007 PMC2374786

[B72] Strader L. C. Monroe-Augustus M. Rogers K. C. Lin G. L. Bartel B. (2008b). Arabidopsis iba response5 suppressors separate responses to various hormones. Genetics 180, 2019–2031. 10.1534/genetics.108.091512 18832358 PMC2600939

[B73] Sun H. Charles C. H. Lau L. F. Tonks N. K. (1993). MKP1(3CH134), an immediate early gene product, is a dual specificity phosphatase that dephosphorylates MAP kinase *in vivo*. Cell 75, 487–493. 10.1016/0092-8674(93)90383-2 8221888

[B74] Tamnanloo F. Damen H. Jangra R. Lee J. S. (2018). MAP KINASE PHOSPHATASE1 controls cell fate transition during stomatal development. Plant Physiol. 178, 247–257. 10.1104/pp.18.00475 30002258 PMC6130035

[B75] Tang Q. Guittard-Crilat E. Maldiney R. Habricot Y. Miginiac E. Bouly J. P.. (2016). The mitogen-activated protein kinase phosphatase PHS1 regulates flowering in *Arabidopsis thaliana*. Planta 243, 909–923. 10.1007/s00425-015-2447-5 26721646

[B76] Tiwari S. B. Hagen G. Guilfoyle T. (2003). The roles of auxin response factor domains in auxin-responsive transcription. Plant Cell 15, 533–543. 10.1105/tpc.008417 12566590 PMC141219

[B77] Tiwari S. B. Hagen G. Guilfoyle T. J. (2004). Aux/IAA proteins contain a potent transcriptional repression domain. Plant Cell 16, 533–543. 10.1105/tpc.017384 14742873 PMC341922

[B78] Uhrig R. G. Labandera A. M. Moorhead G. B. (2013). Arabidopsis PPP family of serine/threonine protein phosphatases: many targets but few engines. Trends Plant Sci. 18, 505–513. 10.1016/j.tplants.2013.05.004 23790269

[B79] Ulm R. Ichimura K. Mizoguchi T. Peck S. C. Zhu T. Wang X.. (2002). Distinct regulation of salinity and genotoxic stress responses by Arabidopsis MAP kinase phosphatase 1. EMBO J. 21, 6483–6493. 10.1093/emboj/cdf646 12456655 PMC136950

[B80] Ulm R. Revenkova E. Sansebastiano G. Bechtold N. Paszkowski J. (2001). Mitogen-activated protein kinase phosphatase is required for genotoxic stress relief in Arabidopsis. Genes Dev. 15, 699–709. 10.1101/gad.192601 11274055 PMC312655

[B81] Walia A. Lee J. S. Wasteneys G. Ellis B. (2009). Arabidopsis mitogen-activated protein kinase MPK18 mediates cortical microtubule functions in plant cells. Plant J. 59, 565–575. 10.1111/j.1365-313X.2009.03895.x 19392697

[B82] Wang C. Li J. Yuan M. (2007). Salt tolerance requires cortical microtubule reorganization in Arabidopsis. Plant Cell Physiol. 48, 1534–1547. 10.1093/pcp/pcm123 17906320

[B83] Ward Y. Gupta S. Jensen P. Wartmann M. Davis R. J. Kelly K. (1994). Control of MAP kinase activation by the mitogen induced threonine/tyrosine phosphatase PAC-1. Nature 367, 651–654. 10.1038/367651a0 8107850

[B84] Whittington A. T. Vugrek O. Wei K. J. Hasenbein N. G. Sugimoto K. Rashbrooke M. C.. (2001). MOR1 is essential for organizing cortical microtubules in plants. Nature 411, 610–613. 10.1038/35079128 11385579

[B85] Widmann C. Gibson S. Jarpe M. B. Johnson G. L. (1999). Mitogen-activated protein kinase: conservation of a three-kinase module from yeast to human. Physiol. Rev. 79, 143–180. 10.1152/physrev.1999.79.1.143 9922370

[B86] Yamakawa H. Katou S. Seo S. Mitsuhara I. Kamada H. Ohashi Y. (2004). Plant MAPK phosphatase interacts with calmodulins. J. Biol. Chem. 279, 928–936. 10.1074/jbc.M310277200 14573600

[B87] Yoo J. H. Cheong M. S. Park C. Y. Moon B. C. Kim M. C. Kang Y. H.. (2004). Regulation of the dual specificity protein phosphatase, DsPTP1, through interactions with calmodulin. J. Biol. Chem. 279, 848–858. 10.1074/jbc.M310709200 14570888

[B88] Zaidi I. Ebel C. Belgaroui N. Ghorbel M. Amara I. Hanin M. (2016). The wheat MAP kinase phosphatase 1 alleviates salt stress and increases antioxidant activities in Arabidopsis. J. Plant Physiol. 193, 12–21. 10.1016/j.jplph.2016.01.011 26927025

[B89] Zaïdi I. Ebel C. Touzri M. Herzog E. Evrard J. L. Schmit A. C.. (2010). TMKP1 is a novel wheat stress responsive MAP Kinase phosphatase localized in the nucleus. Plant Mol. Biol. 73, 325–338. 10.1007/s11103-010-9617-4 20204675

